# Bacterial DNAemia in Older Participants and Nonagenarian Offspring and Association With Redox Biomarkers: Results From MARK-AGE Study

**DOI:** 10.1093/gerona/glac154

**Published:** 2022-08-02

**Authors:** Robertina Giacconi, Patrizia D’Aquila, Marco Malavolta, Francesco Piacenza, Alexander Bürkle, María Moreno Villanueva, Martijn E T Dollé, Eugène Jansen, Tilman Grune, Efstathios S Gonos, Claudio Franceschi, Miriam Capri, Daniela Gradinaru, Beatrix Grubeck-Loebenstein, Ewa Sikora, Wolfgang Stuetz, Daniela Weber, Olivier Toussaint, Florence Debacq-Chainiaux, Antti Hervonen, Mikko Hurme, P Eline Slagboom, Christiane Schön, Jürgen Bernhardt, Nicolle Breusing, Talbot Duncan, Giuseppe Passarino, Dina Bellizzi, Mauro Provinciali

**Affiliations:** Advanced Technology Center for Aging Research, IRCCS INRCA, Ancona, Italy; Department of Biology, Ecology and Earth Sciences (DIBEST), University of Calabria, Rende, Italy; Advanced Technology Center for Aging Research, IRCCS INRCA, Ancona, Italy; Advanced Technology Center for Aging Research, IRCCS INRCA, Ancona, Italy; Molecular Toxicology Group, Department of Biology, University of Konstanz, Konstanz, Germany; Molecular Toxicology Group, Department of Biology, University of Konstanz, Konstanz, Germany; Human Performance Research Centre, Department of Sport Science, University of Konstanz, Konstanz, Germany; Centre for Health Protection, National Institute for Public Health and the Environment, Bilthoven, The Netherlands; Centre for Health Protection, National Institute for Public Health and the Environment, Bilthoven, The Netherlands; Department of Molecular Toxicology, German Institute of Human Nutrition Potsdam-Rehbruecke (DIfE), Nuthetal, Germany; University of Potsdam, Institute of Nutritional Science, Nuthetal, Germany; Department of Physiological Chemistry, Faculty of Chemistry, University of Vienna, Vienna, Austria; National Hellenic Research Foundation, Institute of Biology, Medicinal Chemistry and Biotechnology, Athens, Greece; Department of Experimental, Diagnostic and Specialty Medicine, Alma Mater Studiorum, University of Bologna, Bologna, Italy; Institute of Information Technologies, Mathematics and Mechanics, Lobachevsky University, Nizhniy Novgorod, Russia; Department of Experimental, Diagnostic and Specialty Medicine, Alma Mater Studiorum, University of Bologna, Bologna, Italy; Interdepartmental Center—Alma Mater Research Institute on Global Challenges and Climate Change, University of Bologna, Bologna, Italy; Ana Aslan National Institute of Gerontology and Geriatrics, Bucharest, Romania; Faculty of Pharmacy, Department of Biochemistry, Carol Davila University of Medicine and Pharmacy, Bucharest, Romania; Research Institute for Biomedical Aging Research, University of Innsbruck, Innsbruck, Austria; Laboratory of the Molecular Bases of Ageing, Nencki Institute of Experimental Biology, Polish Academy of Sciences, Warsaw, Poland; Institute of Nutritional Sciences, Department of Food Biofunctionality, University of Hohenheim, Stuttgart, Germany; Department of Molecular Toxicology, German Institute of Human Nutrition Potsdam-Rehbruecke (DIfE), Nuthetal, Germany; University of Potsdam, Institute of Nutritional Science, Nuthetal, Germany; URBC-NARILIS, University of Namur, Namur, Belgium; URBC-NARILIS, University of Namur, Namur, Belgium; The Faculty of Medicine and Health Technology, Tampere University, Tampere, Finland; The Faculty of Medicine and Health Technology, Tampere University, Tampere, Finland; Department of Molecular Epidemiology, Leiden University Medical Centre, Leiden, The Netherlands; BioTeSys GmbH, Esslingen, Germany; BioTeSys GmbH, Esslingen, Germany; Department of Applied Nutritional Science/Dietetics, Institute of Nutritional Medicine, University of Hohenheim, Stuttgart, Germany; Unilever Science and Technology, Bedford, UK; Department of Biology, Ecology and Earth Sciences (DIBEST), University of Calabria, Rende, Italy; Department of Biology, Ecology and Earth Sciences (DIBEST), University of Calabria, Rende, Italy; Advanced Technology Center for Aging Research, IRCCS INRCA, Ancona, Italy

**Keywords:** Aging, Blood bacterial DNA, Dysbiosis, Inflammation, Longevity

## Abstract

Aging and age-related diseases have been linked to microbial dysbiosis with changes in blood bacterial DNA concentration. This condition may promote chronic low-grade inflammation, which can be further aggravated by antioxidant nutrient deficiency. Low plasma carotenoids are associated with an increased risk of inflammation and cellular damage and predict mortality. However, no evidence is yet available on the relationship between antioxidants and the blood bacterial DNA (BB-DNA). Therefore, this study aimed to compare BB-DNA from (a) GO (nonagenarian offspring), (b) age-matched controls (Randomly recruited Age-Stratified Individuals from the General population [RASIG]), and (c) spouses of GO (SGO) recruited in the MARK-AGE project, as well as to investigate the association between BB-DNA, behavior habits, Charlson Comorbidity Index (CCI), leucocyte subsets, and the circulating levels of some antioxidants and oxidative stress markers. BB-DNA was higher in RASIG than GO and SGO, whereas GO and SGO participants showed similar values. BB-DNA increased in smokers and males with CCI ≥ 2 compared with those with CCI ≤ 1 within RASIG. Moreover, BB-DNA was positively associated with lymphocyte, neutrophil, and monocyte counts, but not with self-reported dietary habits. Higher quartiles of BB-DNA were associated with low lutein and zeaxanthin and elevated malondialdehyde plasma concentrations in RASIG. BB-DNA was also positively correlated with nitric oxide levels. Herein, we provide evidence of a reduced BB-DNA in individuals from long-living families and their spouses, suggesting a decreased microbial dysbiosis and bacterial systemic translocation. BB-DNA was also associated with smoking, CCI, leukocyte subsets, and some redox biomarkers in older participants.

The gastrointestinal tract and other epithelial surfaces such as the skin, oral cavity, and lung mucosa host a complex ecosystem of bacteria (1,2). Although the colonization of specific body sites in contact with the external environment by microorganisms is well described and known (3), the existence of microbial populations in other sterile compartments, such as the blood, is a relatively new concept. Previous studies suggest that the presence of bacteria in the blood is a consequence of translocation from other body sites, particularly the gastrointestinal tract, the oral cavity, and the respiratory tract (4–6). With regards to the precise location of microorganisms inside human blood, current evidence suggests that microorganisms can survive inside erythrocytes and leukocytes (7), and in healthy individuals, most bacterial DNA (94%) has been found to be localized within the buffy coat (5).

Recent studies show age-related differences in microbial DNA profiles present in the blood of healthy humans and changes in microbial community composition with aging (8) and with the onset of age-related diseases (5,9,10).

Gut barrier function alterations can occur as an effect of microbial dysbiosis and immune activation, causing bacterial translocation in the bloodstream with possible increment of total blood bacterial DNA (BB-DNA), amplifying the immune-inflammatory responses and promoting the pathophysiology of noninfectious disorders such as obesity, type 2 diabetes, depression, irritable bowel syndrome, cardiovascular, and chronic kidney diseases (11–14).

Although there is evidence that microbial dysbiosis can promote age-associated inflammation (9,15–17), there are no studies investigating BB-DNA in longevity models. Deficiencies in antioxidant nutritional factors may accentuate chronic inflammation (18). For example, low plasma levels of carotenoids have been associated with a higher risk of inflammation and cellular damage (19) with altered intestinal barrier function (20) and may predict mortality in the older adult population (19).

Furthermore, despite the proven antimicrobial activity of some antioxidants such as carotenoids (21), tocopherols (22), and vitamin C (23), no study has investigated a possible relationship between concentrations of circulating antioxidants and BB-DNA.

Therefore, the objective of this study is to compare BB-DNA in GO (offspring of nonagenarians), community-dwelling older adults (Randomly recruited Age-Stratified Individuals from the General population [RASIG]), and spouses of GO (SGO) recruited in the MARK-AGE project, as well as to investigate the association among BB-DNA, behavior habits (diet, smoking, alcohol consumption, and physical activity), Charlson Comorbidity Index (CCI), and oxidant and antioxidant biomarkers.

## Materials and Methods

### Study Population, Recruitment, Data, and Blood Collection

In the present study, we measured BB-DNA in peripheral blood from 1 130 participants recruited during MARK-AGE cross-sectional study (24,25). Participants were in the age range of 54–75 years and came from 8 different European countries. The population studied consisted of 799 RASIG (mean age 64.1 ± 6.1), 212 GO (GEHA offspring; mean age 64.6 ± 5.3), 119 SGO (spouses of GO; mean age 65.3 ± 5.1). GO comprised participants born from a long-living parent belonging to a family with long-living sibling(s), such as the “90+ sib-pairs” recruited within the framework of the EU Integrated Project GEHA, and designated GEHA offspring (GO).

Seropositivity for HIV, HBV (except seropositivity by vaccination), and HCV represented exclusion criteria. Details of the recruitment procedures and the collection of anthropometric, clinical, and demographic data and behavior habits, as well as details of laboratory parameter assays have already been reported (26–28). Comorbidities were evaluated using the CCI (29).

Anticoagulated whole blood, obtained by phlebotomy after overnight fasting, was collected. Prepared samples of plasma, peripheral blood mononuclear cells, and whole blood from the various recruitment centers were shipped to the MARK-AGE Biobank located at the University of Hohenheim, Stuttgart, Germany, on dry ice. From the Biobank, coded samples were subsequently sent to the different laboratories on dry ice, where they were stored at −80°C until use (27).

### 16S rRNA Quantification by Real-Time qPCR

DNA was extracted from 300 µL of whole blood biobanked samples using QIAamp DNA Blood mini kit (Qiagen GmbH, Hilden, Germany) according to the manufacturer’s instructions.

Details in the methodology of 16S rRNA quantification are previously reported (30). Briefly, highly sensitive and specific universal primers targeting the V3–V4 hypervariable region of the bacterial 16S rDNA were used in real-time qPCR reactions to quantify the 16S rRNA gene copy number in DNA samples (31). The PCR mixture (20 µL) consisted of 20 ng of DNA, SensiFAST SYBR Hi-ROX Mix 1× (Bioline, London, UK), and 0.4 µM of the following primers: For 5′-TCCTACGGGAGGCAGCAGT-3′ and Rev 5′-GGACTACCAGGGTATCTAATCCTGTT-3′.

The thermal profile used for the reaction included a heat activation of the enzyme at 95°C for 2 minutes, followed by 40 cycles of denaturation at 95°C for 15 seconds and annealing/extension at 60°C for 60 seconds, followed by melt analysis ramping at 60–95°C. All measurements were taken in the log phase amplification. Standard curves obtained using a 10-fold dilution series of bacterial DNA standards (Femto bacterial DNA quantification kit, Zymo research) ranging from 0.0002 to 2 pg were routinely run with each sample set and compared with previous standard curves to check for consistency between runs. Amplicon quality was ascertained by melting curves. Amplifications of samples and standard dilutions were performed in triplicate on the StepOne Real-Time PCR System (Applied Biosystems by Life Technologies, Waltham, MA). BB-DNA levels were expressed as nanogram per milliliter of whole blood and were calculated by normalizing the absolute quantities of BB-DNA of each sample to their dilution factors and to the volume of starting blood used for the extraction.

A series of controls both in silico and in vitro was performed to exclude artifacts from sample manipulation, reagent contamination, and nonspecific amplifications. The primers were checked for possible cross-hybridization with genes from eukaryotic and mitochondrial genomes using the database similarity search program BLAST. The BLAST search results showed no hits, thus confirming the specificity of primers for the bacterial 16S rRNA as previously reported (31). Separate working areas for real-time PCR mix preparation, template addition, and performing the PCRs were used, and all experimental procedures were performed under a laminar flow hood by using dedicated pipettes, filter-sealed tips, and plasticware guaranteed to be DNA free. Negative controls, in which ultrapure water instead of DNA was added, were also run in each plate.

### Determination of Total Glutathione and Total Free Cysteine in Whole Blood

Total glutathione and total free cysteine in whole blood were measured by RP-HPLC with UV-Vis detection after reduction and modifications as previously reported (32).

### Determination of Ascorbic Acid, Nitric Oxide, and Uric Acid in Plasma

Plasma ascorbic acid and uric acid were analyzed by RP-HPLC and UV detection after reduction with tris-(2-carboxyethyl)-phosphine. The details of the method have been previously reported (32). The concentration of the oxidized metabolites of NO was determined with Nitric Oxide Assay Kit (Abcam, ab 65328), which provides an accurate measure of total nitrate/nitrite reflecting nitric oxide amount in samples. The detection limit of the assay is approximately 1 nmol nitrite/well.

### Determination of Carotenoids, Tocopherols, and Retinol in Plasma

The carotenoids (α-carotene, β-carotene, β-cryptoxanthin, lutein, zeaxanthin, and lycopene), α-tocopherol, ϒ-tocopherol, and retinol were analyzed in plasma and determined simultaneously by RP-HPLC with UV and fluorescence detection as previously described (33).

### Malondialdehyde and Protein Carbonyl Analysis in Plasma

Plasma malondialdehyde was determined by RP-HPLC coupled with fluorescence detection after derivatization with thiobarbituric acid as previously described (32). The determination of plasma protein carbonyls was performed by a sensitive ELISA method as previously reported (34).

### Plasma Isoprostanes

The levels of plasma isoprostanes were detected by a Human 8-epi-prostaglandin F2 alpha (8-iso-PGF2α) time-resolved fluorescence immunoassay on an AutoDELFIA (PerkinElmer).

### Statistical Analysis

Participant characteristics were reported as mean ± *SEM* or percentages for continuous and categorical variables, respectively. For continuous variables, the normal distribution was verified by the 1-sample Kolmogorov–Smirnov test. All the variables not normally distributed were log-transformed. Differences between groups were checked by the 1-way analysis of variance (ANOVA) for continuous variables and Pearson’s χ ^2^ test for categorical variables. ANOVA (after correction for age, sex, countries, smoke habit, and CCI) was used to evaluate differences in BB-DNA levels among GO, SGO, and RASIG or differences in redox parameters among bacterial DNA quartiles. Generalized linear models were also performed to analyze the effect of dietary and lifestyle habits on BB-DNA in RASIG. Multivariate linear regression analysis, with enter method, was performed to evaluate the association between leucocyte subsets and BB-DNA in RASIG participants after sex stratification and adjusting for age and country. A linear regression analysis, using the stepwise method, was also carried out to explore the main predictors of bacterial DNA. The variables included were as follows: age, sex, country, body mass index, oxidative stress markers, and antioxidant nutrients.

The level of statistical significance was set at α ≤ .05.

All the analyses were performed using the SPSS/Win program (version 22.0; SPSS Inc., Chicago, IL).

## Results

### Characteristics of the MARK-AGE Population

Table 1 shows the main characteristics of the RASIG, GO, and SGO populations. All participants had similar age, whereas SGO had a lower percentage of females than males compared with GO and RASIG. Moreover, no significant differences were found in the examined laboratory parameters such as red blood cell number, white blood cell count, number of neutrophils, lymphocytes, monocytes and platelets, albumin, CRP, lipid serum levels, fasting glucose, hemoglobin A1c, and creatinine serum levels. No significant differences were observed in the frequency of cardiovascular diseases, diabetes, depressive symptoms, chronic obstructive pulmonary disease, osteoporosis, and hypothyroidism among groups.

**Table 1.** Characteristics of Participants Selected From the Whole MARK-AGE Population for Circulating Bacterial DNA Analysis

	RASIG	GO	SGO	*p*-Value
	*n* = 799	*n* = 212	*n* = 119	
Age (y)	64.06 ± 0.22	64.64 ± 0.36	65.34 ± 0.47	NS
Females (*n*, %)	413 (51.7)	126 (59.4)	53 (44.5)	.026
RBC (×10^6^/μL)	4.71 ± 0.02	4.71 ± 0.03	4.76 ± 0.04	NS
WBC (×10^3^/μL)	5.97 ± 0.06	6.14 ± 0.14	6.41 ± 0.21	NS
Neutrophils (×10^3^/μL)	3.32 ± 0.04	3.33 ± 0.10	3.49 ± 0.15	NS
Lymphocytes (×10^3^/μL)	1.94 ± 0.26	2.06 ± 0.62	2.11 ± 0.91	NS
Monocytes (×10^3^/μL)	0.48 ± 0.01	0.49 ± 0.01	0.50 ± 0.02	NS
Platelets (×10^3^/μL)	230.0 ± 2.0	232 ± 5.0	221 ± 7.3	NS
Albumin (g/dL)	4.04 ± 0.01	4.02 ± 0.03	3.99 ± 0.04	NS
CRP (μg/L)	2.14 ± 0.12	2.75 ± 0.30	2.98 ± 0.45	NS
TC (mmol/L)	5.73 ± 0.04	5.59 ± 0.09	5.71 ± 0.14	NS
HDL (mmol/L)	1.53 ± 0.01	1.50 ± 0.04	1.43 ± 0.05	NS
LDL (mmol/L)	3.36 ± 0.03	3.52 ± 0.08	3.28 ± 0.12	NS
TG (mmol/L)	1.30 ± 0.03	1.27 ± 0.07	1.63 ± 0.10	NS
FG (mmol/L)	5.40 ± 0.04	5.21 ± 0.10	5.14 ± 0.15	NS
HbA1c (%)	6.07 ± 0.02	6.05 ± 0.06	6.06 ± 0.08	NS
Creatinine (μmol/L)	74.2 ± 0.5	75.3 ± 1.2	75.2 ± 1.8	NS
Cardiovascular diseases	90 (11.3 %)	19 (9.0%)	10 (8.4%)	NS
Hypertension	260 (32.5%)	50 (23.6%)*	38 (31.9%)	.041
Diabetes	75 (9.4%)	16 (7.5%)	10 (8.4%)	NS
Depressive symptoms	106 (13.3%)	25 (11.8%)	9 (7.6%)	NS
COPD	57(7.1%)	15 (7.1%)	4 (3.4%)	NS
Osteoporosis	84 (10.5%)	16 (7.5%)	6 (5.0%)	NS
Hypothyroidism	72 (9%)	14 (6.6%)	7 (5.9%)	NS

*Notes:* COPD = chronic obstructive pulmonary disease; CRP = C-reactive protein; FG = fasting glucose; GO = nonagenarian offspring; HbA1c = hemoglobin A1c; HDL = high-density lipoprotein cholesterol; NS = nonsignificant; LDL = low-density lipoprotein cholesterol; RASIG = Randomly recruited Age-Stratified Individuals from the General population; RBC = red blood cells; SGO = spouses of GO; TG = triglycerides; TC = total cholesterol; WBC = white blood cells. Data are reported as mean ± *SEM*.

*p < .05 versus RASIG.

The laboratory parameter analysis was adjusted for age, sex, countries.

GO participants showed a lower prevalence of hypertension when compared with RASIG. Dietary and lifestyle habits in RASIG, GO, and SGO population are reported in Supplementary Table S1. No significant differences were observed on smoking habit, alcohol consumption, and physical activity among groups. Differences in the consumption of vegetables, meat, dairy products, eggs, and brown and white bread were found among RASIG, GO, and SGO participants.

### BB-DNA Levels in GO, SGO, and RASIG Participants and Their Relation With CCI, Dietary, and Lifestyle Habits

**Figure 1.**
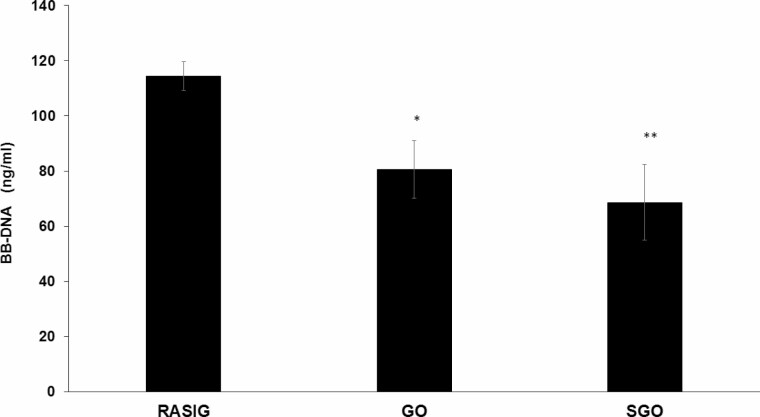
Blood bacterial DNA in peripheral blood from GO, SGO, and RASIG participants. RASIG showed significantly higher BB-DNA levels when compared with the other study groups. GO and SGO participants displayed similar BB-DNA levels. ANCOVA correcting for age, countries, sex, smoke habit, and CCI was applied; data are reported from the model adjusted mean ± *SEM*; *p < .05 when compared with GO; **p < .01 when compared with SGO. BB-DNA = blood bacterial DNA; CCI = Charlson Comorbity Index; GO = nonagenarian offspring; RASIG = Randomly recruited Age-Stratified Individuals from the General population; SGO = spouses of GO.

BB-DNA levels were significantly higher in RASIG compared with GO and SGO participants (Figure 1, *p* < .001), whereas GO and SGO participants showed similar values. Analyzing differences within-participant groups subdivided by sex, RASIG maintained a higher BB-DNA levels only with respect to GO in males and females (Supplementary Table S2).

**Figure 2.**
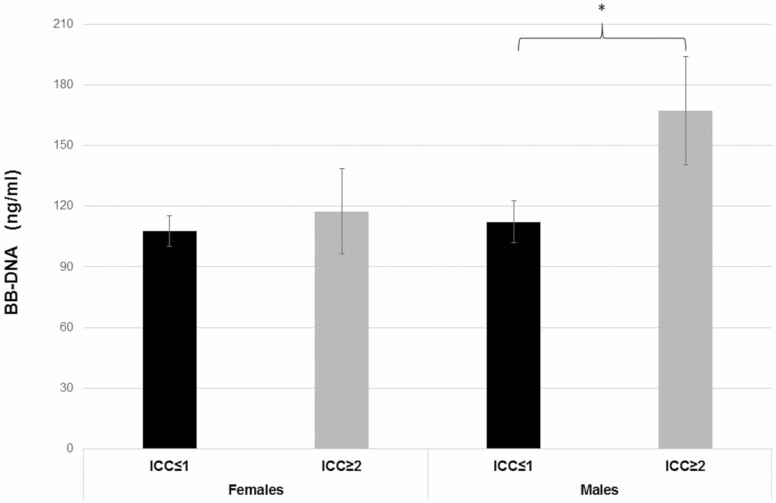
BB-DNA levels in relation to CCI in RASIG population stratified by gender. Males with CCI ≥ 2 showed increased BB-DNA levels compared with males with CCI ≤ 1. *p < .05. ANCOVA stratifying by gender and correcting for age, smoke habit, and countries was applied. BB-DNA = blood bacterial DNA; CCI = Charlson Comorbity Index.

BB-DNA levels were also evaluated in relation to CCI in RASIG population stratified by sex. Characteristics of RASIG population in relation to CCI are reported in Supplementary Table S3. BB-DNA levels increased in males with CCI ≥ 2 compared with those with CCI ≤ 1 (Figure 2, *p* < .05), whereas no significant differences were observed in females. We have also evaluated the influence of dietary and lifestyle habits on BB-DNA in RASIG population. Generalized linear models evidenced that current smokers had higher levels of BB-DNA than former or never smokers in males and only versus never smokers in females (Supplementary Table S4 and Supplementary Figure S1). A lack of association was observed between BB-DNA and self-reported dietary habits, alcohol consumption, or physical activity.

### Multivariate Stepwise Linear Regression Analysis for Antioxidant and Oxidant Parameters Independently Associated With BB-DNA Levels in the RASIG Sample

Independent biomarkers associated with BB-DNA levels (dependent variable) were identified by a linear regression analysis using a stepwise method (Table 2). BB-DNA levels were positively associated with nitric oxide levels (β coefficient = 0.105; *p* < .01) and negatively associated with zeaxanthin plasma levels (β coefficient = −0.082; *p* < .05). No association of BB-DNA was found with age, sex, country, ascorbic acid, total glutathione, α-carotene, β-carotene, β-cryptoxanthin, lutein, and lycopene, α-tocopherol, ϒ-tocopherol and retinol, malondialdehyde, protein carbonyls, total free cysteine, uric acid, and isoprostanes.

**Table 2.** Multivariate Stepwise Linear Regression Analysis for Antioxidant and Oxidant Parameters Independently Associated With BB-DNA Levels in the RASIG Sample

Model		Unstandardized Coefficients		Standardized Coefficients	*p*-Value
		*B*	*SE*	β	
1	NO	0.228	0.080	0.102	.005
2	NO	0.235	0.080	0.105	.004
	Zeaxanthin	−0.141	0.062	-0.082	.024

*Notes:* BB-DNA = blood bacterial DNA; NO = nitric oxide; RASIG = Randomly recruited Age-Stratified Individuals from the General population.

### Association Between BB-DNA, Lymphocyte, Monocyte, and Neutrophil Counts After Sex Stratification in RASIG Population

Multivariate linear regression analysis was performed to evaluate the association between leucocyte subsets and BB-DNA in RASIG participants after sex stratification (Supplementary Figure S2). BB-DNA was positively associated with lymphocyte (male β coefficient = 0.114, *p* < .05; female β coefficient = 0.180, *p* < .01) and neutrophil counts (male β coefficient = 0.224, *p* < .001; female β coefficient = 0.153, *p* < .01) in both sexes. Monocyte count was significantly associated with BB-DNA only in males (male β coefficient = 0.126, *p* < .05; female β coefficient = 0.076, *p* = .136).

### Quartiles of BB-DNA Levels in Relation to Oxidant and Antioxidant Parameters in the RASIG Participants

**Figure 3.**
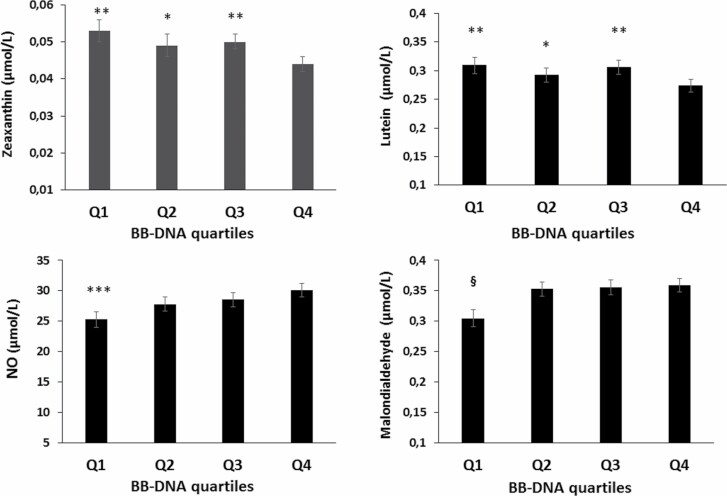
Relationship between BB-DNA quartiles and oxidant and antioxidant parameters in RASIG population. The fourth quartile (Q4) of BB-DNA levels showed the lowest plasma levels of zeaxanthin and lutein when compared with the other quartiles. Nitric oxide was diminished in the first quartile compared with Q3 and Q4 quartiles. The first quartile (Q1) of BB-DNA showed lower levels of malondialdehyde with respect to the fourth quartile. ANCOVA correcting for age, countries, sex, smoke habit, and CCI was applied. ^§^*p* < .05 as compared with Q3 and Q4. ***p < .05 when compared with Q3 and Q4. **p < .01 when compared with Q4. *p < .05 when compared with Q4. BB-DNA = blood bacterial DNA; CCI = Charlson Comorbity Index; RASIG = Randomly recruited Age-Stratified Individuals from the General population.

RASIG sample was divided into quartiles of BB-DNA levels, and the association with oxidant and antioxidant parameters was evaluated. The fourth quartile (Q4) of BB-DNA levels was associated with lower plasma levels of lutein and zeaxanthin when compared with the other quartiles (Figure 3, *p* < .05). The first quartile (Q1) of circulating BB-DNA showed lower levels of plasma malondialdehyde when compared with the fourth quartile (Figure 3, *p* < .05). Moreover, the first quartile of BB-DNA displayed lower NO values with respect to Q3 and Q4 quartiles (Figure 3, *p* < .05). No significant association was found with the other oxidant and antioxidant parameters (Supplementary Table S5).

## Discussion

The presence of the circulating bacterial DNA in blood has been associated with pathological conditions such as diabetes, cardiovascular diseases, chronic kidney disease, obesity, and microbial dysbiosis (8,11–13). Gut dysbiosis is increased in aged people, and it is characterized by microbial composition changes (35), with a reduction of commensals such as Bacteroides, Bifidobacterium, and Firmicutes and an expansion of opportunistic bacteria (34). These proliferating microorganisms can induce chronic low-grade inflammation and promote the onset of age-related degenerative diseases (36,37).

We previously measured BB-DNA levels in RASIG participants. Although we did not find a difference in circulating BB-DNA levels with age (range 35–75 years), we found an association with free fatty acids, insulin, and glucose levels (30), suggesting that persistent high levels of BB-DNA in the bloodstream might predispose to metabolic syndrome or diabetes. Although a causal relationship between increased BB-DNA and the onset of metabolic syndrome or diabetes has not been fully demonstrated, there is evidence showing that insulin resistance has been implicated in impairment of intestinal barrier integrity and increased gut permeability (38,39).

To the best of our knowledge, this is the first study that analyses the differences in BB-DNA levels present in blood of older participants, nonagenarian offspring, and their spouses, as well as the association of DNA amount with oxidative and antioxidant biomarkers.

GO participants had lower bacterial DNAemia than RASIG, suggesting an improved intestinal barrier function and reduced inflammaging (40). It has been shown that persistent antigenic stimulation by an increased pathogen load may be responsible for immunosenescence, leading to increased morbidity and mortality in the older adults (41).

In centenarian offspring, the immune system is more preserved and their proinflammatory status more reduced when compared with older adults without centenarian parents (42). Therefore, the lower DNAemia in GO than RASIG is consistent with this evidence. However, we found no higher anti-inflammatory markers in GO than in RASIG, but only reduced adiponectin levels in SGO females compared with the other 2 participant classes (Supplementary Table S6).

Interestingly, GO and SGO showed similar values of BB-DNA levels probably due to sharing a common environment and lifestyle factors, as also suggested in previous results from MARK-AGE project (43–45).

Herein, we analyzed the differences in certain lifestyle factors such as smoking, alcohol consumption, physical activity, and dietary habits among the participant groups and assessed whether these factors were associated with BB-DNA levels.

Differences were only found in self-reported dietary habits, but not in lifestyle in RASIG compared with GO and SGO, who had similar behavior habits.

Surprisingly, no significant association between self-reported dietary habits and BB-DNA levels was found, although diet affects gut microbiome composition and intestinal barrier integrity (46,47). This fact could be due to the limitation of using a self-reported dietary questionnaire that represents an underaccurate estimation of nutrient consumption compared with measures of nutrient intake, which avoid systematic bias.

Among lifestyle factors, only smoking affects BB-DNA levels and current smokers showed increased bacterial DNAemia when compared with nonsmokers. This result was expected, as the effects of smoking on gut dysbiosis and intestinal permeability changes have already been described (48).

Previous evidence demonstrates that host genetic background influences gut microbiome composition (43,49), whereas the environmental factors such as smoking, exercise, and diet only partially explain changes in the microbiome (43,49).

Many studies conducted in long-lived individuals and their offspring have demonstrated an increased abundance of bacterial species which are considered as potentially beneficial bacteria and linked to body mass index, immunomodulation, and healthier metabolic profile and healthy homeostasis (35,49,50). On the other hand, many commensal bacteria produce useful metabolites that preserve gut barrier integrity (51). It has been demonstrated that cohabitation, especially in spouse pairs, entails a sharing of microbiome composition at the species level (43). On this basis, we can hypothesize that GO have a favorable microbiome composition and preserved intestinal permeability, resulting in reduced BB-DNA compared with RASIG. In addition, GO participants, sharing the same environment and having similar behavior habits (diet, smoking, physical activity) as shown in our data, can pass on to their spouses their own microbiome, likely associated with improved intestinal integrity, and thus explaining the similar BB-DNA levels between GO and SGO.

Of note is also the finding that BB-DNA increases in males with more comorbidities (CCI ≥ 2). As shown in Supplementary Table S3, male participants with CCI ≥ 2 are characterized by a cardiovascular risk profile due to the presence of dyslipidaemia, hyperglycemia, overweight, and subclinical systemic inflammation as suggested by the higher Cu/Zn ratio levels (Supplementary Table S3) (44) and by the increased proinflammatory cytokines observed in a subgroup of male RASIG participants (Supplementary Table S7). These results are consistent with previous evidence that demonstrated as bacterial translocation in the circulation, likely due to altered intestinal barrier, can affect the inflammatory state, contributing to the onset and progression of cardiovascular diseases (11). There are many causes that can affect intestinal barrier permeability including hyperglycemia, increased alcohol consumption, chronic liver disease, alterations in epithelial stem cell turn over, mucus layer thickness, and tight junctions disruption (52). Moreover, innate or adaptive immune dysfunction that occurs with aging can also contribute to the translocation of microbes. The commensal microbiota play a crucial role in reinforcing the gut barrier by preventing colonization by pathogens and producing useful metabolites such as short-chain fatty acids, which promote epithelial health and integrity. Therefore, overgrowth and alterations in the diversity of the intestinal bacterial populations (dysbiosis) can lead to intestinal inflammation and gut barrier alteration (51). It has also been observed a positive association was also observed between BB-DNA and neutrophil, lymphocyte, and monocyte counts, which was partly described in our previous study (30) and which is consistent with recent findings on the implication of gut microbiota in immune cell modulation (53).

Our findings regarding the relationship between BB-DNA levels and NO are concordant with previous evidence showing a positive association between NO blood levels and BB-DNA abundance in cirrhotic patients (54). Circulating BB-DNA triggers an immune response inducing secretion of proinflammatory cytokines that activate the inducible form of NO synthase (iNOS) and release NO with the induction of hemodynamic disturbances (55). NO overproduction by iNOS may lead to decreased endothelial viability and may contribute to tissue damage through both direct cytotoxic effects and the interaction with reactive oxygen intermediates with detrimental effects on the gut barrier, increasing the intensity of bacterial translocation (56,57). Therefore, elevated circulating BB-DNA could induce NO release, and, at the same time, NO overproduction could promote bacterial translocation. Accordingly, GO had lower plasma NO levels than RASIG that in part could explain the reduced presence of BB-DNA levels in the bloodstream (Supplementary Table S8). However, further investigations are needed to better understand these phenomena in aging.

In this study, higher quartiles of BB-DNA were associated with lower levels of certain carotenoids such as lutein and zeaxanthin and higher malondialdehyde plasma concentrations in RASIG, suggesting that an increased bacterial translocation could also depend on an altered redox homeostasis in these participants. In fact, previous evidence shows higher levels of oxidative stress biomarkers in RASIG when compared with GO and different plasma concentrations of some antioxidants (32). Becasue carotenoids have antioxidant and anti-inflammatory properties, improve gut immune function (58), and influence gut microbiome diversity and composition (46), their higher intake and bioavailability may protect intestinal barrier function and prevent gut dysbiosis (47).

Our study has some limitations. First, the 16S microbiome analysis does not discriminate between the presence of just microbial DNA fragments and viable microbes. Second, our study lacked the measurement of markers associated with gut permeability such as zonulin and a characterization of the blood microbiome composition. Third, this study does not clarify whether there are causal relationships between redox changes and increased BB-DNA levels; thus, future studies are needed to explain these aspects.

## Conclusion

In summary, our study provides the first evidence of a reduced BB-DNA presence in the bloodstream of individuals from long-living families and also in their spouses, advocating a decreased microbial dysbiosis and bacterial systemic translocation. Bacterial DNAemia increases in smokers and in males with 2 or more comorbidities, and it is also associated with leukocyte subset counts and some redox biomarkers in older participants, suggesting a role for redox imbalance in promoting bacterial translocation. Our study provides basic evidence for further investigations that can elucidate blood microbiome implications in healthy longevity and age-related diseases.

## Supplementary Material

glac154_suppl_Supplementary_Material

## Data Availability

The data sets used and analyzed during the current study are available from the authors upon reasonable request and with permission of the MARK-AGE Consortium.
