# Supplementary material for: Bacterial DNAemia in Older Participants and Nonagenarian Offspring and Association With Redox Biomarkers: Results From MARK-AGE Study
Source: J Gerontol A Biol Sci Med Sci. 2022 Aug 2;78(1):42–50. doi: 10.1093/gerona/glac154 (PMC9879758; doi:10.1093/gerona/glac154)
Supplement: glac154_suppl_Supplementary_Material [file glac154_suppl_supplementary_material.docx]

**Bacterial DNAemia in older subjects and nonagenarian offspring and association with redox biomarkers: results from MARK-AGE Study.**

**Robertina Giacconi** ^1Ω^**, Patrizia D’Aquila^2^** ^Ω^**, Marco Malavolta^1^, Francesco Piacenza^1^, Alexander Bürkle^3^, María Moreno Villanueva^3,4^, Martijn E. T. Dollé^5^, Eugène Jansen^5^, Tilman Grune^6,7,8^, Efstathios S. Gonos^9^, Claudio Franceschi^10,11^, Miriam Capri^10^,^12^, Daniela Gradinaru^13^, Beatrix Grubeck-Loebenstein^14^, Ewa Sikora^15^,** [**Wolfgang Stuetz**](https://pubmed.ncbi.nlm.nih.gov/?sort=date&size=20&term=Stuetz+W&cauthor_id=34662556)**^16^****,**[**Daniela Weber**](https://pubmed.ncbi.nlm.nih.gov/?sort=date&size=20&term=Weber+D&cauthor_id=34662556)**^6,7^, Olivier Toussaint*^17^, Florence Debacq-Chainiaux^17^, Antti Hervonen^18^, Mikko Hurme^18^, P. Eline Slagboom^19^, Christiane Schön^20^, Jürgen Bernhardt^20^, Nicolle Breusing^21^, Talbot Duncan^22^**, **Giuseppe Passarino^2^, Dina Bellizzi^2§°^, Mauro Provinciali^1§°^**

^1^ Advanced Technology Center for Aging Research, IRCCS INRCA, Ancona, Italy

^2^Department of Biology, Ecology and Earth Sciences (DIBEST), University of Calabria, 87036 Rende, Italy.

^3^ Molecular Toxicology Group, Department of Biology, Box 628, University of Konstanz,

78457 Konstanz, Germany

^4^ Human Performance Research Centre, Department of Sport Science, Box 30, University

of Konstanz, 78457 Konstanz, Germany.

^5^ Centre for Health Protection, National Institute for Public Health and the Environment, PO Box 1, 3720 BA Bilthoven, The Netherlands.

^6^ Department of Molecular Toxicology, German Institute of Human Nutrition Potsdam-

Rehbruecke (DIfE), 14558 Nuthetal, Germany

^7^ University of Potsdam, Institute of Nutritional Science, Nuthetal, Germany.

^8^ Department of Physiological Chemistry, Faculty of Chemistry, University of Vienna, Vienna, Austria.

^9^National Hellenic Research Foundation, Institute of Biology, Medicinal Chemistry and

Biotechnology, Athens, Greece

^10^ Department of Experimental, Diagnostic and Specialty Medicine, Alma Mater Studiorum, University of Bologna, Bologna, Italy.

^11^ Institute of Information Technologies, Mathematics and Mechanics, Lobachevsky University, Nizhniy Novgorod, Russia.

^12^Interdepartmental Center - Alma Mater Research Institute on Global Challenges and Climate Change - University of Bologna, Bologna, Italy

^13^ Ana Aslan National Institute of Gerontology and Geriatrics, Bucharest, Romania; Carol Davila University of Medicine and Pharmacy, Faculty of Pharmacy, Department of Biochemistry, Bucharest, Romania

^14^ Research Institute for Biomedical Aging Research, University of Innsbruck, Rennweg,

10, 6020 Innsbruck, Austria

^15^ Laboratory of the Molecular Bases of Ageing, Nencki Institute of Experimental Biology,

Polish Academy of Sciences, 3 Pasteur street, 02-093 Warsaw, Poland

^16^ Institute of Nutritional Sciences, Dept of Food Biofunctionality, University of Hohenheim, 70593, Stuttgart, Germany.

^17^ URBC-NARILIS, University of Namur, Rue de Bruxelles, 61, Namur, Belgium

^18^ The Faculty of Medicine and Health Technology, Tampere University 33014 Tampere, Finland

^19^ Department of Molecular Epidemiology, Leiden University Medical Centre, Leiden, The

Netherlands

^20^ BioTeSys GmbH, Schelztorstr. 54-56, 73728 Esslingen, Germany

^21^ Department of Applied Nutritional Science/Dietetics, Institute of Nutritional Medicine,

University of Hohenheim, 70599Stuttgart, Germany.

^22^ Unilever Science and Technology, Beauty and Personal Care B50 Tony James Buliding

Colworth Science Park Sharnbrook, Bedford, MK44 1LQ, United Kingdom

* deceased

^Ω^ These authors have contributed equally to this work and share first authorship

^§^ These authors have contributed equally to this work and share last authorship

^°^ Correspondence to:

Robertina Giacconi, Advanced Technology Center for Aging Research, IRCCS INRCA, Ancona, Italy, Phone: +390718004213, email: r.giacconi@inrca.it

Mauro Provinciali, Advanced Technology Center for Aging Research, IRCCS INRCA, Ancona, Italy, Phone: +390718004210, email: m.provinciali@inrca.it

| **Table S1. Dietary and lifestyle habits in RASIG, GO and SGO population**   \|  \|  \| **RASIG** \| **GO** \| **SGO** \| **P value** \| \| --- \| --- \| --- \| --- \| --- \| --- \| \| **Vegetables** \| **<1 serv./day** \| 284 (37.1%) \| 36 (17.9%) \| 18 (15.8%) \| P<0.0001 \| \| **= 1serv./day** \| 360 (47.1%)** \| 124 (61.7%) \| 80 (70.2%) \| \| **≥ 2serv./day** \| 121 (15.8%) \| 41 (20.4%) \| 16 (14.0%) \| \| **Fruit** \| **< 1serv./day** \| 216 (28.2%) \| 43 (21.4%) \| 27 (23.7%) \| NS \| \| **= 1serv./day** \| 336 (43.9%) \| 85 (42.3%) \| 54 (47.4%) \| \| **≥ 2serv./day** \| 231 (27.8%) \| 73 (36.3%) \| 33 (28.9%) \| \| **Meat** \| **≤ 1 serv./wk** \| 83 (10.8%) \| 18 (9.0%) \| 2 (1.8%) \| P<0.001 \| \| **2-6 serv./wk** \| 601 (78.6%)** \| 138 (68.7%) \| 75 (65.8%) \| \| **≥ 7 serv./wk** \| 81 (10.6%) \| 45 (22.4%) \| 37 (32.5%)° \| \| **Dairy Products** \| **< 1 serv./wk or never** \| 103 (13.5%) \| 34 (16.9%) \| 16 (14.0%) \| P<0.05 \| \| **= 1-6 serv./wk** \| 229 (29.9%) \| 38 (18.9%) \| 24 (21.1%) \| \| **≥ 1 serv./day** \| 433(56.6%) \| 129 (64.2%) \| 74 (64.9%) \| \| **Eggs** \| **< 1 serv./wk or never** \| 352 (46.0%) \| 77 (38.3%) \| 38 (33.3%) \| P<0.05 \| \| **≥ 1 serv./wk** \| 413 (54.0%) \| 124 (61.7%) \| 76 (66.7%) \| \| **Fish** \| **< 1 serv./wk or never** \| 313 (40.9%) \| 67 (33.3%) \| 37 (32.5%) \| NS \| \| **≥ 1 serv./wk** \| 452 (59.1%) \| 134 (66.7%) \| 77 (67.5%) \| \| **Brown bread** \| **< 1 serv./wk or never** \| 361 (47.2%) \| 98 (48.8%) \| 51 (44.7%) \| P<0.01 \| \| **= 1-6 serv./wk** \| 188 (24.6%)**°** \| 30 (14.9%) \| 20 (17.5%) \| \| **≥ 7 serv./wk** \| 216 (28.2%) \| 73 (36.3%) \| 43 (37.7%) \| \| **White bread** \| **< 1 serv./wk or never** \| 328 (42.9%) \| 107 (53.2%) \| 62 (54.4%) \| P<0.001 \| \| **= 1-6 serv./wk** \| 216 (28.2%)**°** \| 31 (15.4%) \| 19 (16.7%) \| \| **≥ 7 serv./wk** \| 221 (28.9%) \| 63 (31.3%) \| 33 (28.9%) \| \| **Smoke** \| **never** \| 395 (51,7%) \| 106 (52,7%) \| 53 (46.4%) \| NS \| \| **current** \| 114 (14,9%) \| 24 (11,9%) \| 9 (7.9%) \| \| **previous** \| 255 (33,4%) \| 71 (35,3%) \| 52 (45.6%) \| \| **Alcohol** \| **never** \| 320 (41.8%) \| 103 (51.2%) \| 57 (50.0%) \| NS \| \| **<1/montly** \| 182 (23.8%) \| 41 (20.4%) \| 22 (19.3%) \| \| **<1/weekly** \| 80 (10.5%9 \| 14 (7.0%) \| 8 (7.0%) \| \| **>2-3/weekly** \| 183 (23.9%) \| 43 (21.4%) \| 27 (23.7%) \| \| **Physical activity** \| **Low** \| 523 (68.5%) \| 141 (70.1%) \| 83 (72.8%) \| NS \| \| **Moderate** \| 202 (26.4%) \| 53 (26.4%) \| 26 (22.8%) \| \| **High** \| 39 (5.1%) \| 7 (3.5%) \| 5 (4.4%) \|   Pearson Chi-square test and Post Hoc Tests with Bonferroni adjustment  **p<0.001 vs GO and SGO  ° p<0.05 vs GO  Data were obtained after questionnaires as described in Moreno-Villanueva et al. [26]  **Table S2. Bacterial DNA levels in GO, SGO and RASIG participants subdivided by sex** | | | | | |
| --- | --- | --- | --- | --- | --- | --- | --- | --- | --- | --- | --- | --- | --- | --- | --- | --- | --- | --- | --- | --- | --- | --- | --- | --- | --- | --- | --- | --- | --- | --- | --- | --- | --- | --- | --- | --- | --- | --- | --- | --- | --- | --- | --- | --- | --- | --- | --- | --- | --- | --- | --- | --- | --- | --- | --- | --- | --- | --- | --- | --- | --- | --- | --- | --- | --- | --- | --- | --- | --- | --- | --- | --- | --- | --- | --- | --- | --- | --- | --- | --- | --- | --- | --- | --- | --- | --- | --- | --- | --- | --- | --- | --- | --- | --- | --- | --- | --- | --- | --- | --- | --- | --- | --- | --- | --- | --- | --- | --- | --- | --- | --- | --- | --- | --- | --- | --- | --- | --- | --- | --- | --- | --- | --- | --- | --- | --- | --- | --- | --- | --- | --- | --- | --- | --- | --- | --- | --- | --- | --- | --- | --- | --- | --- | --- | --- | --- | --- | --- | --- | --- | --- | --- | --- | --- | --- | --- | --- | --- | --- | --- | --- |
| SEX |  | Mean | Std. Error | 95% Confidence Interval | |
|  |  |  |  | Lower Bound | Upper Bound |
| Females | RASIG | 108.43 | 6.98 | 94.71 | 122.16 |
|  | GO | 77.56* | 13.62 | 50.81 | 104.32 |
|  | SGO | 70.10 | 28.95 | 13.24 | 126.96 |
| Males | RASIG | 118.23 | 8.80 | 100.93 | 135.52 |
|  | GO | 63.34* | 24.03 | 16.13 | 110.56 |
|  | SGO | 72.047 | 26.08 | 20.79 | 123.29 |
| ANCOVA analysis correcting for age, countries and smoke habit  *p<0.05 as compared to RASIG | | | | | |

**Table S3. Characteristics of RASIG subjects in relation to Charlson comorbidity index and stratified by sex**

|  | **RASIG Females** | | **RASIG Males** | |
| --- | --- | --- | --- | --- |
|  | **CCI≤1** | **CCI≥2** | **CCI≤1** | **CCI≥2** |
| CRP (μg/L) | 2.06±0.15 | 2.38±0.31 | 2.25±0.19 | 2.20±0.32 |
| Cu/Zn ratio | 1.73±0.03 | 1.75±0.05 | 1.43±0.01 | 1.59±0.07^ |
| TC (mmol/L) | 5.91±0.15 | 5.46±0.14 | 1.40±0.05 | 1.64±0.13* |
| HDL (mmol/L) | 1.73±0.022 | 1.56±0.06 | 1.39±0.02 | 1.18±0.05* |
| LDL (mmol/L) | 3.48±0.05 | 3.14±0.12 | 3.39±0.04 | 2.85±0.13* |
| TG (mmol/L) | 1.17±0.03 | 1.46±0.12^ | 1.30±0.05 | 1.64±0.13^ |
| FG (mmol/L) | 5.13±0.04 | 5.71±0.19* | 5.37±0.05 | 6.62±0.35* |
| HbA1c (%) | 6.06±0.02 | 6.47±0.12* | 6.56±0.20 | 6.01±0.03* |
| BMI | 25.9±0.2 | 28.9±0.8* | 26.7±0.19 | 28.95±0.63* |

*p<0.01 as compared to CCI≤1

^ p<0.05 as compared to CCI≤1

ANCOVA analysis correcting for age, countries

**Table S4. Influence of dietary and lifestyle habits on BB-DNA in the RASIG population**

|  | **Wald Chi-Square** | **P value** |
| --- | --- | --- |
| **Smoke** | 19.854 | <0.0001 |
| **Alcohol** | 2.251 | 0.522 |
| **Physical activity** | 0.778 | 0.678 |
| **Vegetables** | 2.175 | 0.337 |
| **Fruit** | 1.023 | 0.600 |
| **Meat** | 1.503 | 0.472 |
| **Dairy Products** | 1.099 | 0.577 |
| **Eggs** | 2.500 | 0.114 |
| **Fish** | 0.077 | 0.782 |
| **Brown bread** | 3.645 | 0.162 |
| **White bread** | 3.331 | 0.189 |

All Generalized linear models included the effect of gender, age, recruitment centre as covariates

Data were obtained after questionnaires as described in Moreno-Villanueva et al. [26]


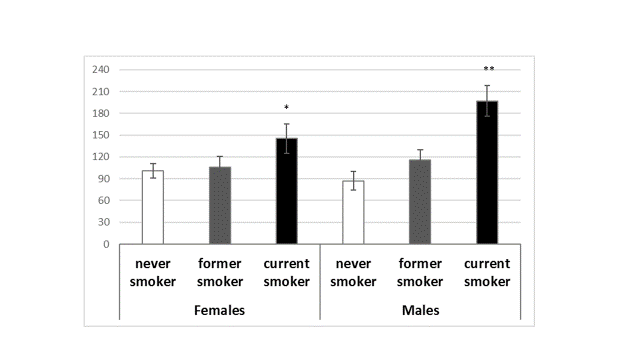


**Fig S1: BB-DNA levels in RASIG population according to smoking habits after sex stratification**

Generalized linear model correcting for age and recruitment center

*p<0.05 as compared to never smoker; **p<0.01 as compared to as compared to former and never smoker


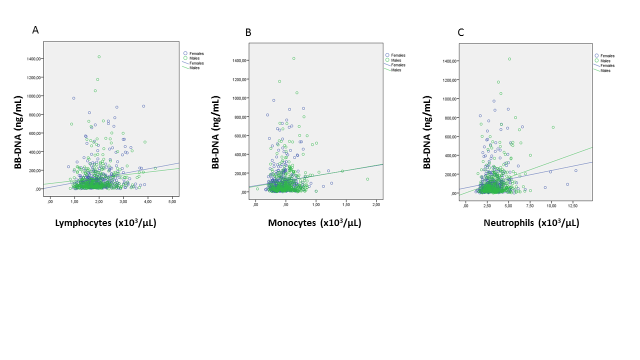


**Fig S2: Association between BB-DNA and lymphocyte, monocyte and neutrophil counts after sex stratification in RASIG population**

Linear regression models adjusting for age and country

A: Males Beta coefficient=0.114, **p= 0.031**; Females Beta coefficient=0.180, **p=0.001**

B: Males Beta coefficient=0.126, **p=0.023**; Females Beta coefficient=0.076, p=0.136

C: Males Beta coefficient=0.224, **p=0.0001** ; Females Beta coefficient=0.153, **p=0.003**

**Table S5. Quartiles of bacterial DNA levels in relation to oxidant and antioxidant parameters in RASIG subjects**

|  | Percentile Group of BB-DNA | Mean | Std. Error | 95% CI | |
| --- | --- | --- | --- | --- | --- |
|  |  |  |  | Lower Bound | Upper Bound |
| **Fat-soluble micronutrients** | |  | | | |
| α-carotene | Q1 | 0.22 | 0.01 | 0.19 | 0.25 |
|  | Q2 | 0.19 | 0.01 | 0.17 | 0.22 |
|  | Q3 | 0.18 | 0.01 | 0.15 | 0.20 |
|  | Q4 | 0.19 | 0.01 | 0.17 | 0.21 |
| β-carotene | Q1 | 0.70 | 0.05 | 0.60 | 0.81 |
|  | Q2 | 0.70 | 0.05 | 0.601 | 0.79 |
|  | Q3 | 0.70 | 0.05 | 0.61 | 0.79 |
|  | Q4 | 0.63 | 0.04 | 0.54 | 0.71 |
| β-cryptoxanthin | Q1 | 0.35 | 0.02 | 0.30 | 0.40 |
|  | Q2 | 0.34 | 0.02 | 0.30 | 0.39 |
|  | Q3 | 0.32 | 0.02 | 0.28 | 0.36 |
|  | Q4 | 0.29 | 0.02 | 0.25 | 0.34 |
| α-tocopherol | Q1 | 29.35 | 0.65 | 28.08 | 30.62 |
|  | Q2 | 30.10 | 0.57 | 28.97 | 31.22 |
|  | Q3 | 29.55 | 0.56 | 28.44 | 30.65 |
|  | Q4 | 29.72 | 0.54 | 28.66 | 30.79 |
| γ-tocopherol | Q1 | 1.50 | 0.70 | 1.41 | 1.68 |
|  | Q2 | 1.67 | 0.06 | 1.56 | 1.79 |
|  | Q3 | 1.52 | 0.06 | 1.41 | 1.64 |
|  | Q4 | 1.61 | 0.06 | 1.50 | 1.72 |
| Retinol | Q1 | 1.74 | 0.03 | 1.67 | 1.80 |
|  | Q2 | 1.82 | 0.03 | 1.76 | 1.88 |
|  | Q3 | 1.75 | 0.03 | 1.69 | 1.81 |
|  | Q4 | 1.74 | 0.03 | 1.69 | 1.80 |
| Lycopene | Q1 | 0.64 | 0.03 | 0.58 | 0.71 |
|  | Q2 | 0.64 | 0.03 | 0.60 | 0.72 |
|  | Q3 | 0.63 | 0.03 | 0.58 | 0.68 |
|  | Q4 | 0.62 | 0.03 | 0.57 | 0.68 |
| **Water-soluble antioxidants** | |  |  |  |  |
| Glutathione | Q1 | 1102.1 | 16.9 | 1068.8 | 1135.4 |
|  | Q2 | 1122.7 | 15.0 | 1093.2 | 1152.2 |
|  | Q3 | 1108.2 | 14.7 | 1079.2 | 1137.2 |
|  | Q4 | 1097.2 | 14.2 | 1069.3 | 1125.0 |
| Cysteine | Q1 | 144.3 | 3.0 | 138.4 | 150.3 |
|  | Q2 | 142.2 | 2.6 | 136.9 | 147.5 |
|  | Q3 | 147.5 | 2.6 | 142.3 | 152.7 |
|  | Q4 | 143.6 | 2.5 | 138.6 | 148.6 |
| Uric acid | Q1 | 44.10 | 0.91 | 42.31 | 45.89 |
|  | Q2 | 45.69 | 0.81 | 44.10 | 47.28 |
|  | Q3 | 46.47 | 0.79 | 44.91 | 48.02 |
|  | Q4 | 45.53 | 0.76 | 44.03 | 47.03 |
| Ascorbic acid | Q1 | 5.08 | 0.23 | 4.63 | 5.53 |
|  | Q2 | 5.03 | 0.20 | 4.63 | 5.43 |
|  | Q3 | 5.41 | 0.20 | 5.01 | 5.80 |
|  | Q4 | 4.79 | 0.19 | 4.42 | 5.17 |
| **Oxidative markers** |  |  | | | |
| Isoprostanes | Q1 | 11.01 | 0.96 | 9.13 | 12.90 |
|  | Q2 | 11.86 | 0.85 | 10.19 | 13.53 |
|  | Q3 | 11.82 | 0.84 | 10.18 | 13.46 |
|  | Q4 | 10.19 | 0.80 | 8.62 | 11.77 |
| Protein carbonyls | Q1 | 0.570 | 0.007 | 0.556 | 0.584 |
|  | Q2 | 0.588 | 0.006 | 0.576 | 0.600 |
|  | Q3 | 0.586 | 0.006 | 0.574 | 0.598 |
|  | Q4 | 0.586 | 0.006 | 0.575 | 0.598 |
| No significant differences in antioxidant or oxidant markers were found among bacterial DNA quartiles  ANCOVA analysis correcting for age, sex and countries | | | | | |

**Table S6. Anti-inflammatory parameters in MARK-AGE study population**

|  | | **RASIG** | **GO** | **SGO** | **P value** |
| --- | --- | --- | --- | --- | --- |
| **Females** | Adiponectin (µg/mL) | 19.12±0.47* | 16.17±0.82 | 13.45±1.24* | P<0.001 |
|  | Vitamin D (nMol/L) | 49.13±0.95 | 45.22±1.65 | 48.02±2.51 | NS |
|  | IL-10 (pg/mL) | 6.78±1.13 | 5.03±1.18 | 6.03±1.80 | NS |
|  | IL-13 (pg/mL) | 6.39±1.18 | 5.94±1.22 | 7.69±1.87 | NS |
| **Males** | Adiponectin (µg/mL) | 11.33±0.33 | 11.25±0.67 | 10.77±0.78 | NS |
|  | Vitamin D (nMol/L) | 49.17±1.05 | 47.29±2.10 | 47.17±2.44 | NS |
|  | IL-10 (pg/mL) | 6.90±0.68 | 9.11±2.61 | 6.08±2.67 | NS |
|  | IL-13 (pg/mL) | 7.77±1.83 | 11.24±2.83 | 6.02±2.90 | NS |

ANCOVA analysis correcting for age, country and BMI

**P<0.01 as compared to GO and SGO

*p<0.05 as compared to GO

Vitamin D was measured in the whole sample following the procedure reported by Stokes et al., 2021 (PMID: 34794520).

Adiponectin was measured in the serum as previously reported Bürkle et al., 2015 (PMID: 25818235).

Cytokine ELISA assays were performed in a subgroup of subjects including 169 RASIG (102 females and 67 males), 84 GO (55 females and 29 males) and 43 SGO (22 females and 21 males) who were random selected.

**Table S7. Pro and anti-inflammatory cytokine plasma levels according to Charlson comorbidity index (CCI) in a subgroup of RASIG**

|  |  | **CCI≤1** | **CCI≥2** | **P value** |
| --- | --- | --- | --- | --- |
| **Females** | IL-1β (pg/mL) | 9.27±1.04 | 3.58±3.16 | NS |
|  | IL-6 (pg/mL) | 7.49±3.542 | 1.56±6.10 | NS |
|  | IL-10 (pg/mL) | 6.46±1.29 | 0.83±2.58 | NS |
|  | TNF-α (pg/mL) | 10.81±2.45 | 3.58±4.91 | NS |
| **Males** | IL-1β (pg/mL) | 9.21±1.66 | 13.73±3.89 | P=0.057 |
|  | IL-6 (pg/mL) | 6.91±2.09 | 15.74±3.95 | P<0.05 |
|  | IL-10 (pg/mL) | 5.93±1.77 | 12.05±3.34 | P=0.072 |
|  | TNF-α (pg/mL) | 10.47±3.19 | 23.61±6.01 | P<0.05 |

ANCOVA analysis correcting for age and country

Cytokine ELISA assays were performed in a subgroup of subjects including 169 RASIG (102 females and 67 males) who were random selected.

**Table S8. NO levels in GO, SGO and RASIG subjects**

|  | Mean | Std. Error | 95% Confidence Interval | |
| --- | --- | --- | --- | --- |
|  |  |  | Lower Bound | Upper Bound |
| **RASIG** | 28.07 | 0.61 | 26.86 | 29.27 |
| **GO** | 24.78** | 1.40 | 22.02 | 27.54 |
| **SGO** | 27.16* | 1.72 | 23.78 | 30.53 |
| ANCOVA analysis correcting for age, sex and countries | | | | |

*p<0.05 as compared to RASIG

**p<0.01 as compared to RASIG
